# Age-Related Changes in Global Motion Coherence: Conflicting Haemodynamic and Perceptual Responses

**DOI:** 10.1038/s41598-018-27803-5

**Published:** 2018-07-03

**Authors:** Laura McKernan Ward, Gordon Morison, Anita Jane Simmers, Uma Shahani

**Affiliations:** 1 Department of Vision Science, Glasgow Caledonian University, 70 Cowcaddens Road, Glasgow, G4 0BA United Kingdom; 2 Department of Engineering, Glasgow Caledonian University, 70 Cowcaddens Road, Glasgow, G4 0BA United Kingdom

## Abstract

Our aim was to use both behavioural and neuroimaging data to identify indicators of perceptual decline in motion processing. We employed a global motion coherence task and functional Near Infrared Spectroscopy (fNIRS). Healthy adults (n = 72, 18–85) were recruited into the following groups: young (n = 28, mean age = 28), middle-aged (n = 22, mean age = 50), and older adults (n = 23, mean age = 70). Participants were assessed on their motion coherence thresholds at 3 different speeds using a psychophysical design. As expected, we report age group differences in motion processing as demonstrated by higher motion coherence thresholds in older adults. Crucially, we add correlational data showing that global motion perception declines linearly as a function of age. The associated fNIRS recordings provide a clear physiological correlate of global motion perception. The crux of this study lies in the robust linear correlation between age and haemodynamic response for both measures of oxygenation. We hypothesise that there is an increase in neural recruitment, necessitating an increase in metabolic need and blood flow, which presents as a higher oxygenated haemoglobin response. We report age-related changes in motion perception with poorer behavioural performance (high motion coherence thresholds) associated with an increased haemodynamic response.

## Introduction

Our ability to perceive movement is perhaps the most crucial of visual processes and vital to our survival in the world. The fundamental purpose of human vision is to enable us to interact with our environment^1^. Our visual system must analyse the entire visual scene, producing an overall or ‘global’ percept by integrating across the visual field, and not simply focusing on a single element. This is commonly referred to as global motion perception. Though physiologically there is relative sparing of the visual cortices in ageing^2,3^, many perceptual processes decline^4^, including motion perception^5–14^. Whilst some types of motion are only moderately affected by age (biological^13,15–17^, radial^13,18–20^), results for translational motion (e.g. up vs. down, left vs. right) are perhaps more widely studied. This motion processing of detection and/or discrimination, has repeatedly shown an age-related decline in perception^5–10,12–14,18,19,21–31^. Many of these behavioural studies focus on very specific stimulus parameters and often include substantial methodological differences. Our focus was the impact of healthy ageing, and as such we employed a simple motion coherence task to study the effect of this, without attempting to reconcile the vast methodological differences in the current literature. Rather than use two extremes of older and younger adult age groups, here we examined the entire spectrum of the adult lifespan from ages 18–85. We employed a translational global motion task (Random-Dot-Kinematogram, RDKs) and functional Near Infrared Spectroscopy (fNIRS) to determine age-related differences in perception across the adult lifespan. Our aim was to use both behavioural and neuroimaging data to investigate any indicators of perceptual decline in motion processing.

There is an increasing evidence base showing that poorer translational global motion perception is associated with healthy ageing^5–14,18–31^. The majority of this research uses RDKs, which effectively isolate motion processing; the stimulus consists of ‘signal’ dots moving in the same direction, and ‘noise’ dots moving randomly. Observers must identify the overall direction of motion from local signal dots, ignoring the noise. When there are more signal dots moving within the array, it is easier to detect the direction of motion, i.e. the image has high motion coherence. Younger adults (typically <30 years old) outperform older adults (typically >60 years old) on these motion tasks, even when differences in visual acuity and blur are controlled for^9^. Low motion coherence thresholds (typically <20%) indicate high performance. With ageing, coherence thresholds increase and task performance decreases. The precise extent of this age-related deterioration of global motion perception is estimated to be between 2–13.5%^8,30^. This wide range is most likely due to the substantial methodological differences in parameter choices between behavioural studies. Some studies have reported that when RDK dots are moving at 1.6, 2.5, approximately 6, 10, and at 28 degrees per second (°/s), older adults demonstrate worse performance than their younger counterparts^5–10,19^. However, other authors have reported no such age-related correlations at 2, 4 or 6 °/s^11,12^. Similarly, there is evidence to suggest that older adults are comparable to younger adults at detecting 100% coherence in motion^12^, and additionally, that age has an advantageous effect when the stimulus aperture size is small^29^. We overcame these inconsistencies by using a large display and long duration of stimulus presentation so that it was easy for every adult (regardless of age) to perceive the stimulus. Our hypothesis was that any differences in global motion coherence thresholds would be the result of ageing. We used translational global motion to provide a simple motion-processing task to determine age-related differences in perception across the adult lifespan.

Physiologically, there is little change across the human lifespan in the visual cortex (in terms of loss of grey matter density^32^ and volume^33^, white matter volume^34^, blood volume, and iron content^35^). Theories of age-related performance should thus be considered in this context; structurally there is little difference in the visual cortices of young and older adults. Yet, an increasing number of neuroimaging studies report functional differences in visual perception between younger and older age groups in line with the dedifferentiation theory of ageing^13,36–41^. This hypothesis suggests that older brains become less functionally distinct (decreased modularity), because highly specialised cortical areas become less specific and show a decrease in selectivity in the processing of stimuli^42^. Biehl *et al*.^13^, recently reported an increased V5 response in older adults compared to younger adults when viewing radial motion stimuli^13^. The age groups presented not only with differential patterns of V5 activation, but also older adults had an additional recruitment of neurons within the right inferior frontal gyrus, supporting the notion of dedifferentiation. Similarly, EEG evidence shows older adults to present with a more topographically distributed P1 motion response in the visual cortex, despite comparable performance to younger adults on the associated behavioural task^43,44^.

There is an increasing recognition of the network of brain areas involved in motion processing, which is not a singular process reliant on just a single visual area. Emerging literature in fMRI, high resolution 7 T fMRI and patient studies demonstrates that motion perception involves multiple brain regions, not solely V5^13,45–53^. Neural correlates of global motion coherence thresholds occur in areas V5, V3a, V6, ventral occipital surface, intraparietal sulcus, and temporal structures^48,50,53–55^. The rationale for this work therefore assumes a more distributed network of motion processing within the brain. Both behavioural and neuroimaging results on ageing in motion suggest the neural correlates of motion processing may not be fixed across the lifespan. To examine age-related changes in motion perception, we measure extrastriate cortical activity approximating V5.

To improve comparability with the literature, we employ both behavioural measures of motion thresholds, and neuroimaging measures of the Haemodynamic Response (HDR) recorded by fNIRS. fNIRS is a noninvasive imaging technique that can measure the visual cortex’s HDR^56^. fNIRS operates by shining wavelengths of light (between 650 nm–950 nm) through fibre-optic emitters. Changes in the light absorbed, scattered, and reflected, are recorded by detectors, and directly relate to changes in the optical properties of blood, more specifically, to the haemoglobin protein (the oxygen carrier). Two wavelengths of light are used to calculate both concentrations of oxygenated- ([HbO]) and deoxygenated-haemoglobin ([HbR]). fNIRS has previously been used to successfully record visual perception in healthy adults^57–74^. This technique offers several advantages as a system to study ageing: reduced sensitivity to motion artefacts, increased participant tolerance to environment, increased inclusion (no magnetic safety contraindications), and lastly the ability to separate [HbO] and [HbR]. Using fNIRS, we captured the response from a large area of the extrastriate cortex that included V5. The specific region of interest, though important, was not critical to inherently capture a NIRS HDR to moving stimuli.

## Results

### Data Analysis

fNIRS data pre-processing was completed with a custom-written MATLAB script, which included the following: normalisation, smoothing and average response calculation. Using a simple subtraction method, all data were normalised with respect to the pre-stimulus baseline (the last 20 seconds of a 60 seconds baseline). This ensured that the normalisation procedure was carried out according to the most stable average response to the control image (baseline fixation cross). A conservative 3-point moving average filter was computed on the normalised data. This low-pass filtered data were then de-trended. The mean of 10 to 20 seconds post-stimulus onset was used, representing the most reliable response to the stimulus^75–77^. This timing allowed the HDR to peak immediately after the stimulus was presented, ensuring the recording of a stable perceptual response. The mean of this global motion response was compared with the baseline (grey screen with white cross) visual stimulation. The robust Median Absolute Deviation (MAD) method of outlier analysis was used. As there were no statistically significant differences between the hemispheres these were averaged to create an overall V5 response.

### Group Descriptives

Group descriptives can be seen in Table 1. There were n = 28 younger adults (mean age of 28 ± 7.3), n = 22 middle-aged adults (mean age of 50 ± 6.9), and n = 23 older adults (mean age of 70 ± 6.9). A multivariate ANOVA showed the expected age-related differences (systolic and diastolic BP, p < 0.05). Pairwise comparisons revealed that both younger and middle-aged adults had lower systolic BP (F_2, 67_ = 8.08, p < 0.01, η^2^ = 0.19) and diastolic BP (F_2, 67_ = 3.64, p < 0.05, η^2^ = 0.09), in contrast to older adults. These group statistics were supported by Pearson’s skipped correlations with 95% CI using MATLAB’s Robust Correlations toolbox^78^. Skipped correlations is a robust method of Pearson’s r using the linear association but ignoring outliers detected by taking into account the overall structure of the data via bootstrapping techniques^78^. Age was significantly correlated with both systolic and diastolic BP (r = 0.41, CI = [0.21 0.61], r = 0.25, CI = [0.04 0.41], respectively). When entering sex as a between-subject factor for each of these outcome measures, there were no statistically significant results, indicating that there were no sex differences in our sample (p > 0.05), unlike a handful or previous studies^18,25,26^. None of these descriptive outcome measures (BP, HR, eCRF) correlated significantly with the other data (behavioural or neuroimaging).

**Table 1:** Group descriptives of each age group with means (SD). BP (mmHg), HR (beats per minute), eCRF (higher scores represent better fitness).

	Younger (<40)	Middle-aged (40–60)	Older (>60)
N	28 (F = 22)	22 (F = 11)	23 (F = 12)
Age	28.1 (7)	49.9 (7)	69.8 (7)
Systolic BP	124 (11)	131 (18)	142 (20)
Diastolic BP	79 (9)	83 (11)	87 (11)
HR	69 (11)	69 (14)	72 (13)
eCRF	12.38 (2.2)	10.51 (4.0)	7.17 (2.7)

### Behavioural Results

**Figure 1:**
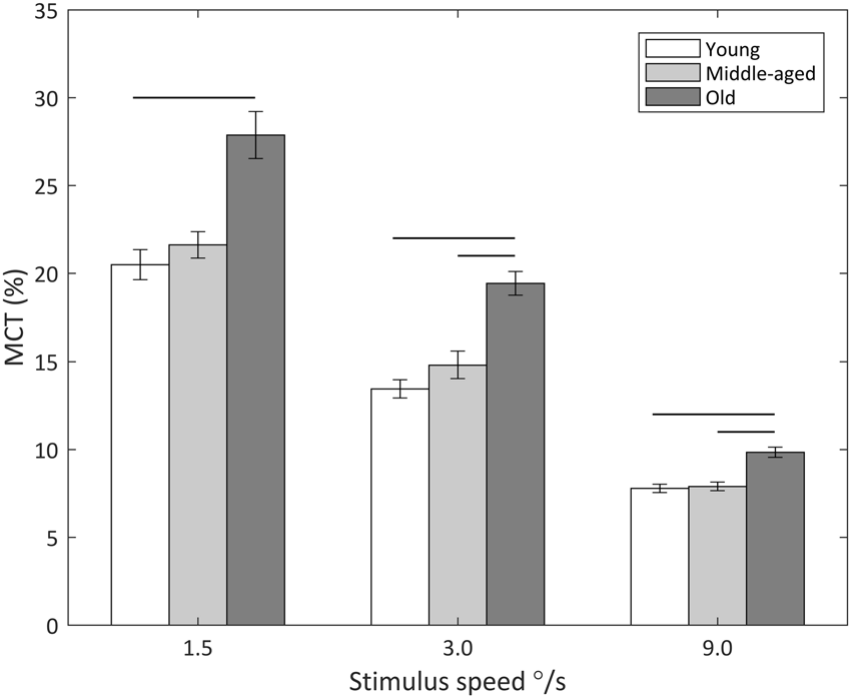
Behavioural response to psychophysical testing: MCTs of the three age groups (younger, middle-aged, older adults), at the three stimuli speeds (1.5, 3, 9°/s). Means and SEM plotted. Lines depict significant differences between the groups (see text, p < 0.05).

**Figure 2:**
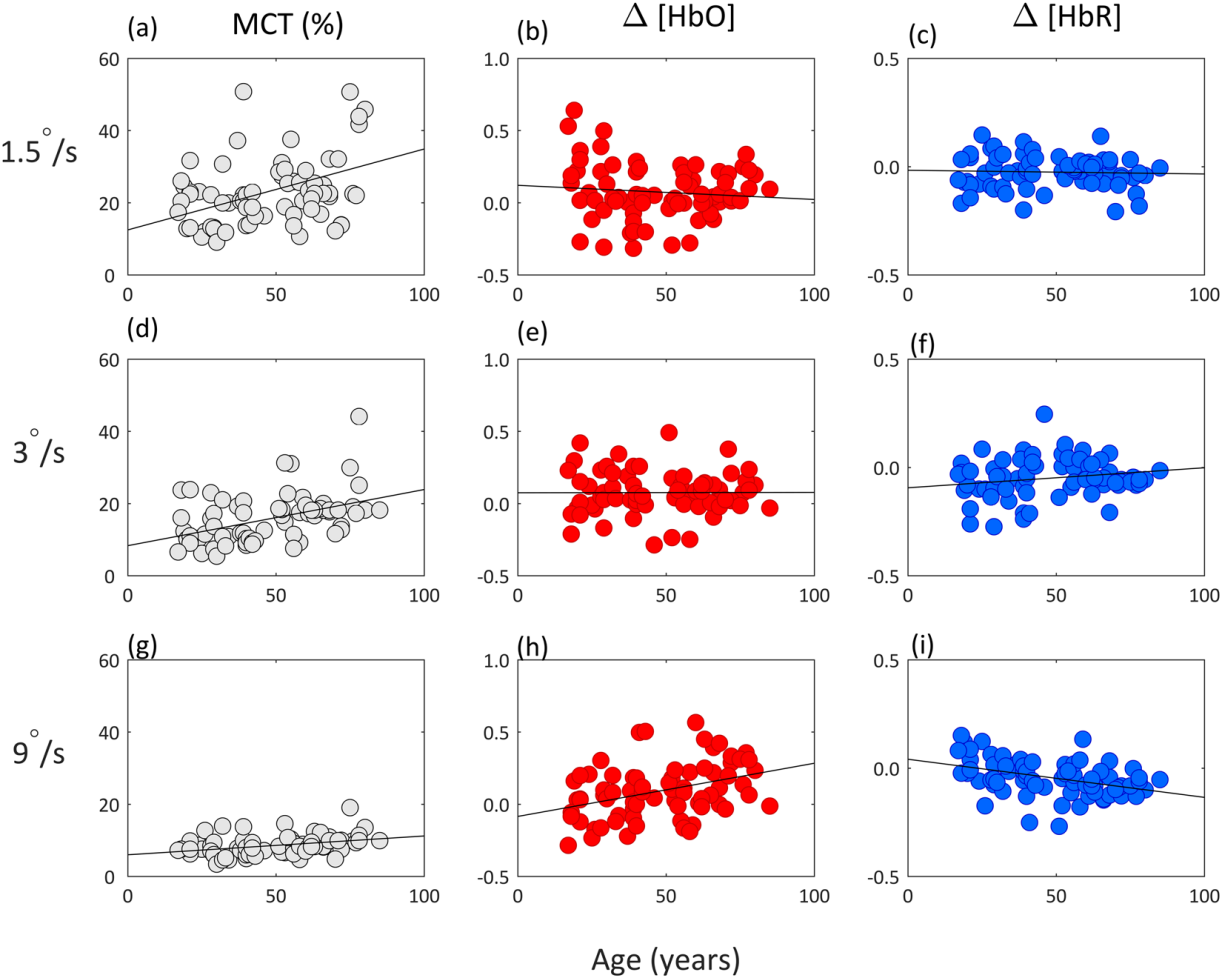
Individual average motion thresholds (MCT % plotted in grey) and HDR to global motion stimulus ([HbO] plotted in red, [HbR] plotted in blue), plotted against age. The first row shows responses to the 1.5°/s stimuli speed, the second shows responses to 3°/s and responses to 9°/s are shown in the bottom row. Statistically significant correlations were found with age and MCTs for the 3 (r = 0.38) and 9°/s stimulus speed (r = 0.37). Correlational results between age and [HbO] found at 9°/s stimulus speed (r = 0.37), for age and [HbR] found at 3 (r = 0.19) and 9°/s (r = −0.41) speeds.

Individual Motion Coherence Threshold (MCT) data showed a robust Pearson’s skipped correlation of age associated with poorer motion perception at 3 (r = 0.38 CI = [0.16 0.57]) and 9°/s stimulus speeds (r = 0.37, CI = [0.18 0.55]) (Fig. 2d,g). Age group differences were assessed using a 3-way ANOVA with speed entered as a within-subject factor and group as a between-subject factor. There were consistent age-related differences for each speed of stimulus: 1.5 (F_2, 72_ = 3.86, p < 0.05, η^2^ = 0.01), 3 (F_2, 72_ = 6.06, p < 0.01, η^2^ = 0.02) and 9°/s (F_2, 72_ = 4.94, p < 0.01, η^2^ = 0.01). Post-hoc independent sample T-tests with adjusted CIs (99.99%) revealed these differences to be mostly between the oldest age group compared to the youngest and middle-aged groups (Fig. 1). The younger adults outperformed the older adults at every stimulus speed: 1.5 (mean difference = −7.38, t_49_ = −2.40, p < 0.05, d_s_ = 0.68), 3 (mean difference = −5.99, t_49_ = −3.58, p < 0.001, d_s_ = 1.01) and 9°/s (mean difference = 2.05, t_49_ = −2.78, p < 0.01, d_s_ = 0.78). The middle-aged adults also performed better than the older adults at 3 (mean difference = −4.63, t_42_ = −2.27, p < 0.05, d_s_ = 0.69) and 9°/s (mean difference = −1.95, t_42_ = −2.54, p < 0.05, d_s_ = 0.77). Perhaps this result (middle-aged adults outperforming older adults) failed to appear for the slower 1.5°/s speed because of the increase in variability of all participants’ thresholds. Overall there was a large effect of stimulus speed as evidenced by a multivariate ANOVA (F_2, 142_ = 125.9, p < 0.001, η^2^ = 0.64). Poorer (and more varied) performance was associated with slower speeds of motion, regardless of age groups, particularly at 1.5°/s. To check if trial length influenced these results, a 3-way multivariate ANOVA was run for each speed, no significant differences were found. This indicates that groups were comparable and MCT responses cannot be attributed to processing speed or tiredness for example, but are indeed reflective of perceptual differences.

### Neuroimaging HDR to Motion

The HDR to motion perception presented as a characteristic fNIRS response with an increase in [HbO] and decrease in [HbR] during motion perception compared to the baseline fixation cross. A 2 × 3 repeated measures ANOVA was conducted with stimulation (baseline, motion) and speed (1.5, 3, 9°/s) as within-subject factors and group as a between-subject factor. Overall, results for [HbO] showed an effect of motion stimulation with an incredibly large effect size (F_1, 64_ = 37.09, p < 0.001, *η*^2^ = *0*.*94*). Similarly, [HbR] also presented with a large main effect of motion stimulation (F_1, 61_ = 56.94, p < 0.001, *η*^2^ = *0*.*91*).

To investigate the effect of age on the motion HDR we ran robust Pearson’s skipped correlations for each [HbO] and [HbR] (Fig. 2). During the faster 9°/s motion stimuli older age was correlated with an increase in [HbO] (r = 0.37 CI = [0.18 0.54]) (Fig. 2h). This is a good effect size with robust confidence intervals demonstrating a clear linear age-related effect to the motion HDR. These individual level results were also found at group level with the ANOVA indicating a small 3-way interaction between stimulation, speed, and age group (F_4, 128_ = 2.98, p < 0.05, η^2^ = 0.08). To determine exactly where group differences occurred, independent T-tests were performed post-hoc with adjusted CIs (99.99%). During both 1.5 and 3°/s motion stimuli, age groups were equivalent in their [HbO] in their HDR. However, during the faster 9°/s motion stimuli there were age-related effects, thus supporting the robust individual correlational results. Older adults showed significantly increased [HbO] responses compared to middle aged (mean difference = −0.12, t_42_ = −2.19, p < 0.05, d_s_ = 0.65) and younger adults (mean difference = −0.20, t_49_ = −4.39, p < 0.001, d_s_ = 1.24). A similar pattern of results was found for [HbR], with Pearson’s robust skipped correlations showing older age to correlate with [HbR] during 3 (r = 0.19, CI = [0.01 0.36]) (Fig. 2f) and 9°/s (r = −0.41, CI = [0.59 0.2]) (Fig. 2i). Once more, group results supported individual level findings with a 3-way interaction between stimulation, speed, and group (F_4, 122_ = 5.57, p < 0.001, η^2^ = 0.15). Adjusted post-hoc T-tests showed group differences at both 3 and 9°/s stimuli, but not at the slower 1.5°/s. There were statistically significant differences between younger and middle-aged adults (mean difference = 0.07, t_47_ = 2.76, p < 0.01, d_s_ = 0.79), and younger and older adults (mean difference = 0.08, t_49_ = 4.74, p < 0.01, d_s_ = 1.33).

There were mixed findings for the two slower speeds of stimulus (1.5°/s, 3°/s). However, results for the fastest stimuli (9°/s) showed robust and distinct age-related effects wherein [HbO] and [HbR] mirrored each other. This HDR to motion presented as a characteristic fNIRS response with an increase in [HbO] and decrease in [HbR] during stimulus presentation compared to the baseline fixation cross. Subsequent age-related changes in motion perception were strongest during the fastest stimulus speed with a dot presentation of 9°/s. Note that these HDR results were in response to a stimulus with high global motion coherence, because trials always began with 100% coherence and the algorithm subsequently adjusted stimulus presentation depending on individuals’ performance (and response speed). There is fMRI evidence of a direct linear relationship between coherence of globally moving stimuli and V5 activation^53,79–81^. Therefore, as this HDR represents such a motion response to stimuli with high coherence, rather than being specific to individuals’ thresholds, it represents a general perceptual HDR to global motion. Overall results can be seen in Table 2 with a summary of the behavioural and neuroimaging results for each speed and age group comparison.

**Table 2:** Summary of both significant and non-significant findings for each statistical test: robust Pearson’s skipped correlations, r values presented with confidence intervals [CI], 3-way ANOVA interaction for stimulation, speed, and group (p values), and the post-hoc Independent samples T-test with adjusted CI’s (99.99%) (p values).

			Behavioural	fNIRS	
			MCT	HbO	HbR
1.5°/s	Skipped correlation	r	0.20 [−0.04 0.41]	−0.09 [−0.33 0.17]	−0.04 [−0.28 0.20]
	3-way ANOVA interaction		0.026^*^	0.022^*^	0.000^**^
	T-test	Y vs M	0.637	0.545	0.636
		Y vs O	0.022^*^	0.982	0.804
		M vs O	0.532	0.423	0.408
3°/s	Skipped correlation	r	0.38 [0.16 0.57]^**^	0.00 [−0.23 0.23]	−0.19 [0.01 0.36]^**^
	3-way ANOVA interaction		0.004^**^	as above	
	T-test	Y vs M	0.453	0.380	0.003^**^
		Y vs O	0.001^**^	0.934	0.152
		M vs O	0.029^**^	0.367	0.039^*^
9°/s	Skipped correlation	r	0.37 [0.18 0.55]^**^	0.37 [0.18 0.54]^**^	−0.41 [0.59 0.2]^**^
	3-way ANOVA interaction		0.01^**^	as above	
	T-test	Y vs M	0.875	0.110	0.008
		Y vs O	0.008^**^	0.000^**^	0.000^**^
		M vs O	0.015^*^	0.034^*^	0.477

*p < 0.05, **p < 0.005.

### Coupling of cerebral oxygenation measures

**Figure 3:**
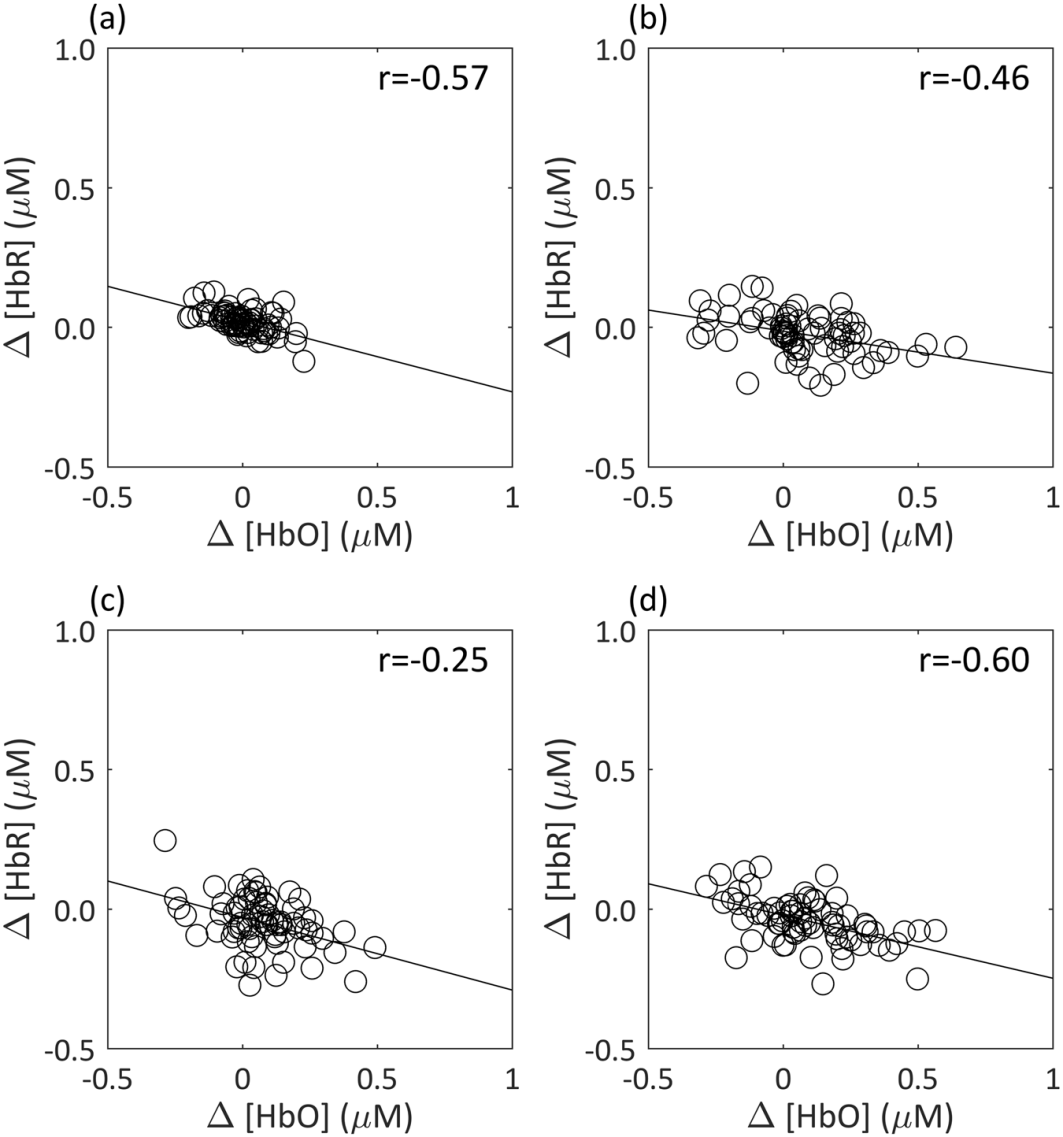
Individual grand average HDR for [HbO] plotted against [HbR] for each (**a**) baseline, (**b**) 1.5°/s, (**c**) 3°/s and (**d**) 9°/s stimulus speeds. Significant correlations demonstrating tight coupling for each (**a**) r = −0.57, (**b**) r = −0.46, (**c**) r = −0.25, and (**d**) r = −0.60.

Pearson’s skipped correlations were performed to investigate the coupling between the cerebral oxygenation measures for each speed of stimuli and baseline response, i.e. relationship between [HbO] and [HbR] during recordings. Regardless of age, there was significant coupling for all experimental manipulations: 1.5 (r = −0.46, CI = [−0.61 − 0.29]), 3 (r = −0.25, CI = [−0.42 − 0.05]), 9 (r = −0.60, CI = [−0.74 − 0.44]), and resting state-responses (r = −0.57, CI = [−0.72 − 0.40]). These results demonstrate tight physiological coupling between [HbO] and [HbR] during the motion task as well as at rest (Fig. 3).

## Discussion

This is the first fNIRS study to investigate global motion perception in a healthy ageing population. We used a global motion task to study the perceptual thresholds and associated physiological changes of the HDR in adults aged 18–85 years old. The expected behavioural results were present with a reduced global motion coherence perception in older adults demonstrated by higher motion coherence thresholds. In contrast to these behavioural findings, the HDR was greater in older adults. When using robust statistical methods, we report greater [HbO] and [HbR] HDR as a function of older age, i.e. there was a linear correlation between age and the motion HDR.

In accordance with previous literature, the psychophysical results show an age-related decline in global motion perception^5–10^. In terms of motion coherence thresholds, i.e. a measure of individually determined motion perception, this decline was much greater than previously reported^30^. The mean difference between young and older adults was 7%, and middle-aged and older adults 6%. Regardless of the speed of the RDK stimulus used, older adults had the highest thresholds (poorest performance). Interestingly, these results are the opposite of Pilz *et al*., (2017) who reported older adults to perform similar to younger adults detecting vertical motion^12^. However, there are a number of methodological differences that may underpin this: the older adult group was slightly younger than the current group, individual MCTs were calculated using the classical method of constant stimuli rather than the current adaptive staircase procedure, and the stimuli contained just under double the number of dots than ours did. The current differences in age groups support previous evidence on decade group thresholds and propose the greatest difference to be with the ‘oldest of old’, namely over the age of 70 years old^7,28^. Similarly, the greatest behavioural differences we report were with the older adults (aged 60–85), compared to younger and middle-aged adults (see Fig. 1). Crucially, what we add here is the correlational data, as our sample included adults aged 18–85 years old. Robust statistics revealed the 3 and 9°/s stimulus speeds were associated with poorer motion perception with increasing age. As Fig. 2 shows, our rigorous methodology ensured that the age-related decline in motion perception could not be attributed to differences in acuity or speed of processing. Particularly noteworthy is the data distribution for the fast stimulus speed (9°/s, Fig. 2g); there is less variability in data distribution compared to the other speeds, which suggests that the results at 9°/s were reliable. Real world implications for this task have been shown by Conlon *et al*., (2015) who reported thresholds at the same speed and showed older adults’ motion coherence thresholds were related to self-reported driving difficulties^82^.

The data provide a clear physiological correlate of motion perception as reflected by the HDR. This is the first study to use FDMD-fNIRS during a motion task in healthy adults providing absolute values of cerebral oxygenation measures. Regardless of speed, all participants showed a distinctive HDR with an increase of [HbO] and decrease of [HbR] during stimulation. This HDR to motion processing and associated statistical effect sizes, show the magnitude of the changes between the baseline fixation cross and subsequent motion stimulation. These results support two previous fNIRS studies using a passively recorded HDR to different motion stimuli (motion illusion^83^ and radial motion^66^), both of which report an increase in [HbO] and decrease in [HbR] during stimulation. The crux of the current findings lies in the differences in responses recorded across the lifespan: both poor motion perception (high motion coherence thresholds) and an increased HDR were modulated by age. We hypothesize that older adults required the recruitment of additional neurons to complete the task successfully. The speculated increased neural recruitment necessitated an increased metabolic need and subsequent increase in blood flow, presenting as higher [HbO] responses to global motion stimulation in older adults. Whilst we acknowledge the spatial limitations of fNIRS, our data show unequivocally, a HDR that is a physiological reflection of global motion perception recorded over the parieto-occipital cortex approximating V5.

The current results fit the dedifferentiation theory of healthy ageing. Visual dedifferentiation has been reported in older adults (compared to younger adults) in both early and late stages of visual processing^38,84^. Both univariate and multivariate studies show an age-related reduction in neural distinctiveness in visual regions in older adults^13,36–41^. For example, Biehl *et al*.^13^, recently showed older adults presented with differential fMRI activation patterns compared to younger adults. Age-related differences in processing radial motion were present with older adults recruiting larger areas of the middle/superior temporal gyrus and additional brain regions compared to younger adults^13^. Similarly, electrophysiological work has shown older adults to present with a greater topographical voltage distribution in response to translational motion compared to younger adults, despite equivalent behavioural performance on the associated task^43^. Our fNIRS data can provide absolute quantification of the HDR in terms of [HbO] and [HbR]. As can be seen in Fig. 3, there is tight physiological coupling between these two measures of cerebral oxygenation, regardless of age. This is in line with previous literature reporting no age-related effects of coupling^85,86^. Here we report an increased HDR as measured by fNIRS in ageing adults in response to seeing a global motion stimulus. We propose that older adults required an additional recruitment of neurons for processing and perceiving global motion.

Event-related processing was not possible in this experimental design. This is a limitation that may easily be addressed in future work where triggering could provide information about the shape of the HDR to specific coherence levels. The HDR recorded was in response to the initial presentation of the motion stimuli, i.e. when the image had high coherence. As individual performance varied, the HDR could not be attributed to a specific coherence level. However, previous neuroimaging evidence indicates a linear correlation between high coherence of moving stimuli and the V5 response^45,53,79–81^. Therefore, we propose our HDR to be a sound reflection of global motion processing.

To conclude, we report an age-related decline in global motion perception thresholds (across all speeds of stimuli used). The fastest speed (9°/s) generated the most robust results for both behavioural and neuroimaging data. Regardless of age, all participants showed a distinctive increase of [HbO] and decrease of [HbR] during visual stimulation. Our novel results show that fNIRS can reliably capture the HDR associated with global motion perception. Moreover, strong vascular coupling between [HbO] and [HbR] was shown regardless of age or experimental manipulation. The significance of this work lies in the age-related differences in the HDR, with an increased HDR from younger, to middle-aged, to older adults. The significant results and large effect sizes reported here relate each of the behavioural and neuroimaging data to be affected as a function of age, in line with the dedifferentiation theory of ageing. We hypothesise that there is an increase in neural recruitment, and therefore a consequent increase in cerebral blood flow, in response to an age-related decline in motion processing.

## Methodology

### Participants

A total of 72 healthy adults between the ages of 18–85 were recruited, and were broadly divided into the following age groups: young adults (n = 28, mean age 28 ± 7.3 range 18–39, 22 females), middle-aged adults (n = 22, mean age 50 ± 6.9, range 40–59, 12 females), and older adults (n = 23, mean age 70 ± 6.9, range 60–85, 12 females). All participants had a Visual Acuity (VA) of at least 6/9 (6/6 equating to ‘normal’ vision) with optical correction where required and had no history of neurological or psychiatric disorders. A short medical history was taken and any current medication noted with none being reported that would influence the cerebral blood flow (e.g. those participants on hypertensive medication would not bias results^87^). Measurements of height, weight, Blood Pressure (BP), and Heart Rate (HR), were taken at rest prior to beginning the main experiment. Estimated Cardiorespiratory Fitness (eCRF) was calculated using an algorithm including sex, body mass index, resting HR, and self-reported physical activity. The eCRF has previously been used with success and reliability providing an accurate estimate of cardiorespiratory fitness (compared to a NASA exercise test) regarding cerebral blood flow^88,89^. Lastly, all participants had a comparable number of years of education within each subgroup and scored >27 on the Mini-Mental State Examination (MMSE) for cognitive screening. Glasgow Caledonian University’s Ethics Committee approved the research protocol, and informed written consent was obtained from all participants prior to testing. All research was performed in accordance with the relevant guidelines and regulations of this ethics committee, conforming to the Declaration of Helsinki.

### Visual Stimuli

**Figure 4:**
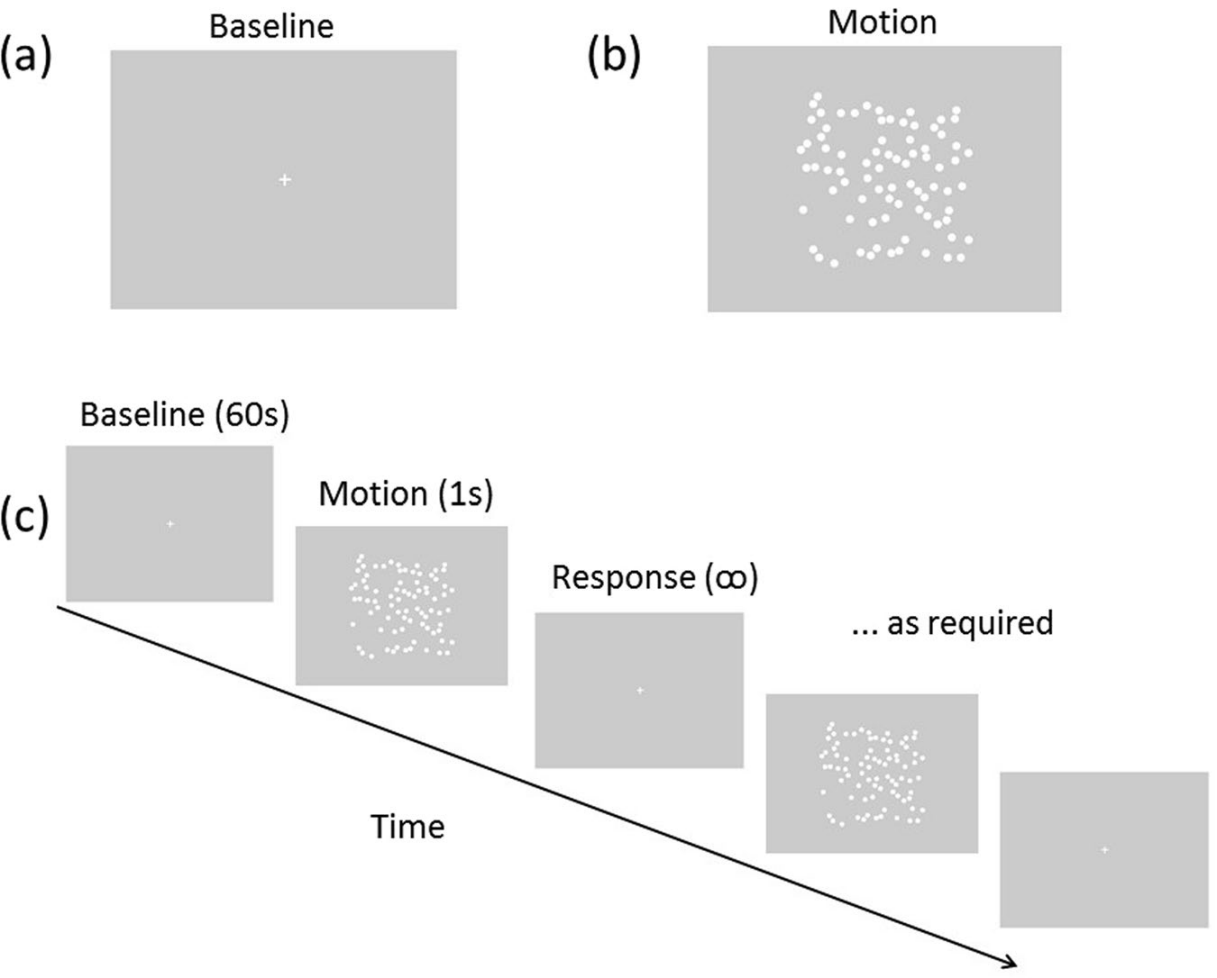
Experimental protocol showing (**a**) the baseline image, (**b**) a static screen-shot of the global motion stimulus, and (**c**) the overall procedure. The global motion discrimination task was continued until the staircase paradigm could calculate an individual’s threshold (i.e. timing varied for each participant).

Participant’s Motion Coherence Threshold (MCT) was assessing using a standard RDK paradigm^90^. The 80 white moving dots (luminance 100 cd/m^2^) were presented within a central square display, on a homogenous grey background (luminance 50 cd/m^2^). The motion array can be seen in Fig. 4b. The direction of signal dots (moving either up or down) had to be detected over random noise dots. Each dot was displayed for 160, 80, or 26.67 ms. Varying the dot duration created apparent motion at 3 stimulus speeds: 1.5, 3, and 9°/second. Dot density, size, brightness, displacement, radius, background brightness, and spatial offset were kept constant. Participants viewed the stimulus at 57 cm, so that 1 screen pixel subtended 0.04°. Before beginning the motion task, participants were presented with a white central fixation cross (Fig. 4a) during which a baseline imaging response of 60 seconds was recorded. A button press started the task, and a motion array did not appear until a response had been recorded to the previous array. Figure 4 depicts the experimental paradigm including the baseline (a) and motion array (b), and the timing of a trial (c).

### Psychophysical Staircase Parameters

Participants completed 3 trials of each speed, in a randomised counterbalanced order, and the mean MCT to each speed was calculated. The MCT is the minimum percentage of signal dots required to successfully determine the global motion direction. A 2AFC adaptive staircase procedure was used that tracked 79.4% correct threshold value. On each staircase reversal, the step size was halved (starting at 16). When this reached 1 dot, the mean of the 6 last reversals was used to calculate the threshold. The final MCT for each speed was calculated as a mean of the 3 trials completed for an accurate estimation of the individual’s threshold. This methodology and stimulus has previously been used to assess the sensitivity of global motion detection in amblyopic adults^90,91^.

### HDR Recording

**Figure 5:**
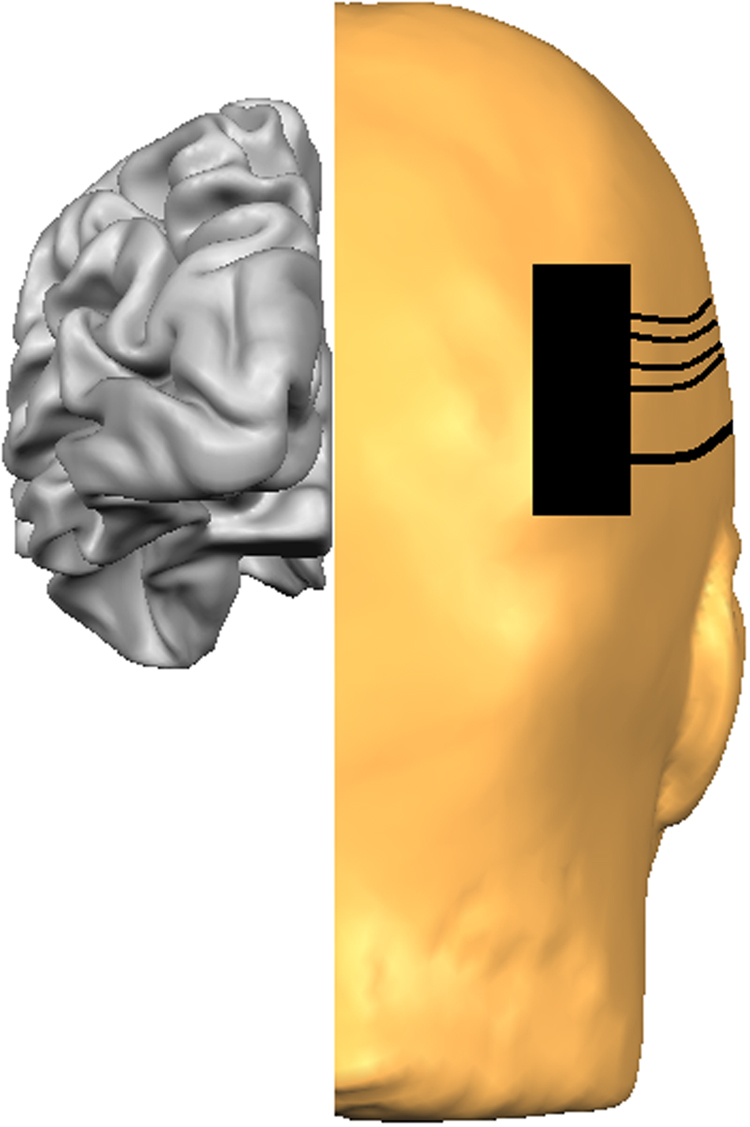
Diagram of fNIRS set-up with the sensor placed over the right hemisphere approximating V5. All recordings were taken from over both right and left parieto-occipital cortices.

A Frequency-Domain Multi-Distance (FDMD) fNIRS system was used with two-channels (OxiplexTS^TM^). This system is frequency modulated at 110 MHz with 1 Hz sampling. Two wavelengths of light (690 nm and 830 nm) are used to calculate changes of [HbO] and [HbR] in absolute concentration (μM/L). This instrumentation has been described in detail elsewhere^76,92,93^. Near infrared light detectors were at fixed distances from the emitters (ranging from 1.9–3 cm) in each of the two silicone sensors. These precise distances were entered into the OxiplexTS software to subsequently calculate cerebral oxygenation levels, using absorption, scattering and phase data and the modified Beer-Lambert Law^56,93^. HDRs to the grey screen (see Fig. 4) were recorded prior to stimulus onset. As the stimuli consisted of moving dots, we recorded over the parieto-occipital cortex, namely V5. During completion of the motion task, fNIRS recordings were taken from over the area thought to coincide with V5’s location (Fig. 5). This approximation was based on an average of previously reported fMRI Talariach coordinates and subsequently converted to the EEG 10–20 International System of Electrode Placement^45,83,94–96^. fNIRS was recorded on a trial-by-trial basis resulting in 9 HDRs per participant (3 speeds × 3 trials).

### Data Availability

The datasets generated during the current study are available from the corresponding author on reasonable request.
